# *In vitro* and *in vivo* Virulence Potential of the Emergent Species of the *Acinetobacter baumannii* (Ab) Group

**DOI:** 10.3389/fmicb.2019.02429

**Published:** 2019-10-24

**Authors:** Clara Cosgaya, Carlos Ratia, Marta Marí-Almirall, Laia Rubio, Paul G. Higgins, Harald Seifert, Ignasi Roca, Jordi Vila

**Affiliations:** ^1^ISGlobal, Hospital Clínic – University of Barcelona, Barcelona, Spain; ^2^Institute for Medical Microbiology, Immunology and Hygiene, University of Cologne, Cologne, Germany; ^3^German Center for Infection Research (DZIF), Bonn, Germany

**Keywords:** *Acinetobacter*, multi-drug resistance, virulence, biofilm formation, *C. elegans*, efflux pumps, oxacillinases

## Abstract

The increased use of molecular identification methods and mass spectrometry has revealed that *Acinetobacter* spp. of the *A. baumannii* (Ab) group other than *A. baumannii* are increasingly being recovered from human samples and may pose a health challenge if neglected. In this study 76 isolates of 5 species within the Ab group (*A. baumannii n* = 16, *A. lactucae n* = 12, *A. nosocomialis n* = 16, *A. pittii n* = 20, and *A. seifertii n* = 12), were compared in terms of antimicrobial susceptibility, carriage of intrinsic resistance genes, biofilm formation, and the ability to kill *Caenorhabditis elegans* in an infection assay. In agreement with previous studies, antimicrobial resistance was common among *A. baumannii* while all other species were generally more susceptible. Carriage of genes encoding different efflux pumps was frequent in all species and the presence of intrinsic class D β-lactamases was reported in *A. baumannii*, *A. lactucae* (heterotypic synonym of *A. dijkshoorniae*) and *A. pittii* but not in *A. nosocomialis* and *A. seifertii*. *A. baumannii* and *A. nosocomialis* presented weaker pathogenicity in our *in vitro* and *in vivo* models than *A. seifertii*, *A. pittii* and, especially, *A. lactucae*. Isolates from the former species showed decreased biofilm formation and required a longer time to kill *C. elegans* nematodes. These results suggest relevant differences in terms of antibiotic susceptibility patterns among the members of the Ab group as well as highlight a higher pathogenicity potential for the emerging species of the group in this particular model. Nevertheless, the impact of such potential in the human host still remains to be determined.

## Introduction

Among the 62 validly named *Acinetobacter* species (List of Prokaryotic Names with Standing Nomenclature^1^, last accessed April 2019), *Acinetobacter baumannii* has, for many years, monopolized our attention. Responsible for bloodstream infections, ventilator-associated pneumonia, wound infections, and meningitis, among other infections, *A. baumannii* excels in its ability to persist and survive in the nosocomial setting (Peleg et al., 2008). In comparison, little is known regarding the closely related members of the so-called *A. baumannii* (Ab) group, such as *Acinetobacter nosocomialis* and *Acinetobacter pittii*, previously known members of the group, but also *Acinetobacter lactucae* (heterotypic synonym of *A. dijkshoorniae*) and *Acinetobacter seifertii*, whose species status has recently been acknowledged (Nemec et al., 2015; Cosgaya et al., 2016). Species other than *A. baumannii* among the Ab group are increasingly being isolated from human specimens, albeit not as frequently as *A. baumannii*, and they also possess an undeniable ability to cause disease (Espinal et al., 2011; Schleicher et al., 2013; Chen et al., 2018; Pailhories et al., 2018; Rocha et al., 2018). Unfortunately, they are often erroneously identified as *A. baumannii* by common phenotypic methods such as biochemical tests and semi-automated identification systems, thus underestimating their true prevalence and clinical relevance. Accurate species identification is achieved by molecular methods, such as ARDRA, *rpoB* and *gyrB* sequence analysis, Pasteur MLST scheme-based identification, whole genome sequencing (Vaneechoutte et al., 1995; Gundi et al., 2009; Diancourt et al., 2010; Higgins et al., 2010b; Fitzpatrick et al., 2016) and, more recently, mass spectrometry (Mari-Almirall et al., 2017).

Antimicrobial resistance studies containing information about *A. lactucae*, *A. nosocomialis*, *A. pittii* and *A. seifertii* are scarce, although existing data support their general susceptibility to most antibiotics (Chuang et al., 2011; Lai et al., 2012; Chen et al., 2018; Pailhories et al., 2018). In contrast, *A. baumannii* is considered a problem pathogen because of its frequently reported multi-drug resistant (MDR) phenotype. This pathogen has developed resistance to all antibiotics, including last resort antibiotics such as carbapenems, tigecycline and colistin (Roca et al., 2012; Nowak et al., 2017). Multi-drug resistance represents one of the best assets of *A. baumannii* and may account for its success in the hospital setting; nonetheless other pathogenic attributes may as well impact its ability to infect the host.

Likewise, there is plenty of information on the virulence traits of *A. baumannii* (Harding et al., 2018) but little is known regarding the other members of the Ab group. Studies regarding the virulence attributes of different *Acinetobacter* species are rare, and usually include only a few isolates (de Breij et al., 2010; Bitrian et al., 2012; Peleg et al., 2012; Kinsella et al., 2017) or are limited to bacteremic isolates from a unique region (Na et al., 2016). Noticeably, none of the abovementioned studies include information about *A. lactucae* and there is just one report that highlights its ability to cause disease and carry genetic determinants conferring carbapenem resistance (Espinal et al., 2015).

A few reports have suggested relevant differences in the clinical outcomes of infections caused by former members of the Ab group (Chuang et al., 2011; Wisplinghoff et al., 2012; Lee et al., 2013; Chusri et al., 2014), suggesting that, in order to understand the clinical relevance and the virulence potential of closely related *Acinetobacter* spp., further studies on susceptibility patterns and pathogenesis need to address them as distinct entities rather than as a homogeneous group. In this work we have gathered a collection of representative isolates from all five known *Acinetobacter* species of the Ab group and compared their antibiotic susceptibility profiles and carriage of resistance determinants as well as their pathogenic potential using both *in vitro* and *in vivo* assays.

## Materials and Methods

### Bacterial Strains, Culture Conditions, and Epidemiologic Characterization

Isolates used in this study belong to *Acinetobacter baumannii* (*n* = 16), *A. lactucae* (*n* = 12), *A. nosocomialis* (*n* = 16), *A. pittii* (*n* = 20) and *A. seifertii* (*n* = 12) (Supplementary Data Sheet S1). Species identification was performed by matrix-assisted laser desorption ionization-time of flight mass spectrometry (Mari-Almirall et al., 2017). The clonal relatedness was established either by repetitive element palindromic PCR (REP-PCR) or by pulsed-field gel electrophoresis (PFGE) of *Apa*I-digested DNA (Seifert et al., 2005). The sequence type (ST) was determined following the Pasteur multilocus sequence typing (MLST) scheme, as previously described (Diancourt et al., 2010). Bacterial cultures were routinely grown on Columbia sheep blood agar (Becton Dickinson, Heidelberg, Germany) at 37°C, unless stated otherwise.

### Antimicrobial Susceptibility Testing and Presence of Acquired Resistance Genes

The minimum inhibitory concentration (MIC) was determined by broth microdilution or gradient diffusion (AB bioMérieux, Solna, Sweeden) in Mueller Hinton II broth (CONDA, Madrid, Spain) or agar plates (Becton Dickinson, Heidelberg, Germany), respectively, and interpreted according to EUCAST guidelines when appropriate (EUCAST clinical breakpoints version 8.0, 2018). As no EUCAST susceptibility breakpoint for ceftazidime is available, the corresponding clinical breakpoint according to CLSI guidelines was used instead (susceptible ≤ 8 μg/mL, resistant ≥ 32 μg/mL; CLSI supplement M100-S28, 2018). The antibiotics tested were amikacin, ceftazidime, ciprofloxacin, colistin, gentamicin, imipenem, meropenem, tigecycline, and tobramycin. Presence of metallo-β-lactamases, *bla*_KPC_, *bla*_OXA–__23__,_ *bla*_OXA–__24__/__40__,_ and *bla*_OXA–__58_ genes was determined by multiplex PCR (Bogaerts et al., 2013) or characterized in previous studies (Supplementary Data Sheet S1).

### Detection of Genes Potentially Associated With Intrinsic Resistance

The presence of genes encoding efflux pumps from the resistance-nodulation-cell division (RND) family (*adeABC*, *adeDE*, *adeFGH*, and *adeIJK*), and intrinsic oxacillinases (OXA) from the class D β-lactamases was evaluated by PCR using the primers listed in Supplementary Table S1. *In silico* analysis of available genomic sequences at NCBI were performed in order to ensure amplification in all the Ab group species, otherwise, species-specific primers were designed. At the time of the analysis, only one *A. seifertii* genome and only two *A. lactucae* genomes were available at the NCBI databases. Intrinsic OXA β-lactamases were analyzed by DNA sequencing and cluster analysis of partial amino acid sequences (residues 9 to 267) using the MEGA version 6 software (Tamura et al., 2013). Briefly, sequences were aligned using MUSCLE (Edgar, 2004) and unrooted phylogenetic trees were constructed using the neighbor-joining method with bootstrap values based on 1000 replicates, with software default settings. When necessary, additional sequences were retrieved from public databases. The partial sequences of allelic variants used for cluster analysis are provided in the Supplementary Data Sheet S2.

### Quantitative Biofilm Formation Assay

Biofilm formation was assessed in non-treated 96-well polystyrene microtiter plates. Bacteria were grown overnight at 37°C with shaking in modified M63 medium consisting of KH_2_PO_4_ (12 g/l), K_2_HPO_4_ (7 g/l), (NH_4_)_2_SO_4_ (2 g/l), adjusted to pH 7 with KOH prior to autoclaving, and supplemented with glucose (0.2% w/v), MgSO_4_ (1 mM) and casaminoacids (0.5% w/v). Overnight cultures were adjusted to an OD_600_ of 0.1 and diluted 1:10 in modified M63 medium. For each isolate four wells were inoculated with 150 μL of the former suspension. Afterward, plates were incubated statically at 28 or 37°C. After 44 h, supernatants were removed with a 20 G needle, wells were washed once with PBS, and biofilm was fixed with methanol 99% for 10 min and evaporated at 65°C for 20 min. Biofilm was stained with crystal violet 2% for 20 min. Glacial acetic acid 33% was used to solubilize the dye and absorbance was recorded at 580 nm. The OD_580_/OD_600_ ratio was used to quantify biofilm formation and to normalize for bacterial growth differences. At least three biological replicas with a covariance for the OD_580_/OD_600_ ratio less than 45% were performed for each isolate. Isolates were considered biofilm producers when the OD_580_/OD_600_ ratio was greater than 1. Appropriate controls were added for all assays and all plates, and consisted of bacteria-free media and the inclusion of *A. baumannii* ATCC 19606 and *A. baumannii* ATCC 17978 bacterial strains as strong and weak biofilm producers, respectively.

### Surface-Associated Motility Assay

Motility plates consisted of tryptone (5 g/L), NaCl (2.5 g/L) and agarose (0.3% w/v) (CONDA, Madrid, Spain). Prior to pouring, media was cooled down to 50°C in a water bath. Twenty mL were dispensed in 90 mm diameter petri dishes and allowed to solidify for 45 min plus an additional 15 min for air-drying in a biosafety cabinet. Then, 1 μL of the same bacterial suspension used for biofilm assays was inoculated onto the center of the surface of the media, plates were sealed with parafilm and incubated at 37°C for 18 h. Isolates tended to grow concentrically from the inoculation point, thus surface-associated motility was recorded as the mean value (cm) of three diameter measurements per plate. Isolates were considered motile when the diameter exceeded 1 cm. In every assay, isolates were tested in duplicate, and at least four biological replicates were performed for all the isolates. In all the assays, *A. baumannii* ATCC 19606 and *A. baumannii* ATCC 17978 were used as non-motile and motile control strains, respectively.

### *Caenorhabditis elegans* Killing Assays

The Fer-15 line of *C. elegans*, fertile at 15°C but not at 25°C and provided by the Caenorhabditis Genetics Center, was used for the infection model. Nematodes were routinely maintained at 15°C and fed with the non-virulent *E. coli* OP50 strain in Nematode Growth Medium agar. Assays were performed using L4 stage worms as previously described (Espinal et al., 2019). Live worms were scored every day for 15 days using a stereomicroscope. Worms were considered dead when unresponsive to touch and immotile for more than 20 s. The lethal time 50% (LT50), that is, the time (days) needed to kill 50% of the worms, of each isolate in each assay was obtained by extrapolation from the sigmoidal regression of the killing curves (*R*^2^ > 0.95) using GraphPad Prism version 5 for Windows (GrapPad Software, La Jolla, CA, United States). At least three biological replicates were performed for each isolate. In all the assays, *E. coli* OP50 was used as a non-virulent control strain.

### Statistical Analysis

All statistical analyses were performed using IBM SPSS Statistics for Windows, version 23 (IBM, Corp., Armonk, NY, United States). Kolmogorov–Smirnov test or Shapiro–Wilk test was used to assess normal distribution when *n* > 30 or *n* < 30, respectively. For the regression models, the Pearson correlation coefficient was determined when the variables followed a normal distribution. Whenever non-normally distributed variables or ordinal variables were analyzed, the Spearman rank correlation coefficient or the Kendall’s τ correlation coefficient were calculated, respectively. The Kruskal–Wallis test was performed to compare the distribution of variables among the different species; *post hoc* analyses were automatically performed by the software. Pairwise differences were compared using Mann–Whitney *U-*test. The Wilcoxon signed rank test was used to compare biofilm formation at 28 and 37°C using quantitative data, whereas the McNemar test was used to compare the proportions of biofilm-producing isolates at both temperatures. For all tests performed, *P*-values < 0.05 were considered to be statistically significant.

### Ethics Statement

The experimental procedures described in this work do not involve the usage of biological samples from humans or animals. Clinical bacterial isolates studied here were obtained from clinical collections at several microbiology labs where they were initially recovered from clinical samples used for microbiological diagnosis. Informed consent was, therefore, not required. The protocol for this study was approved by the Ethics Committee on Clinical Research (CEIC) of the Hospital Clinic de Barcelona (HCB/2014/0499, HCB/2017/0923, and HCB/2017/0833).

## Results

### Antimicrobial Susceptibility Profiles

We have characterized the susceptibility patterns of 76 isolates from 5 different *Acinetobacter* species within the Ab group, as shown in Table 1. Isolates other than *A. baumannii* were usually susceptible to almost all antibiotics tested, with only a few exceptions. The *A. seifertii* isolates from our collection were susceptible to all the agents tested, thus constituting the species with the lowest rates of resistance. Likewise, isolates of *A. nosocomialis* were susceptible to all agents except the aminoglycosides, for which rates of resistance were 25% for gentamicin and 5% for amikacin.

**TABLE 1:** Antimicrobial susceptibility profiles of the Ab group species.

**Antimicrobial**		***A. baumannii* (*n* = 16)**	***A. lactucae*(*n* = 12)**	***A. nosocomialis* (*n* = 16)**	***A. pittii* (*n* = 20)**	***A. seifertii* (*n* = 12)**
Amikacin	MIC_50_	4	0.75	1.5	1.5	1
	MIC_90_	128	1.5	6	4	2
	Range	0.38–>256	0.38–3	1–12	0.75–24	0.5–12
	R(%)	31.3	0	5	0	0
Ceftazidime	MIC_50_	128	3	3	3	4
	MIC_90_	>128	32	4	16	6
	Range	2–>128	2–>256	1.5–4	1.5–>256	2–12
	R(%)^a^	75	25	0	5	0
Ciprofloxacin	MIC_50_	64	0.25	0.0125	0.19	0.25
	MIC_90_	>64	0.38	0.5	0.5	0.38
	Range	0.094–>64	0.19–0.38	0.064–0.75	0.125–>32	0.064–0.75
	R(%)	68.85	0	0	5	0
Colistin	MIC_50_	0.38	0.25	0.19	0.19	0.125
	MIC_90_	1	0.38	0.38	0.5	0.25
	Range	0.38–8	0.19–0.38	0.38–0.5	0.19–1	0.125–0.38
	R(%)	6.3	0	0	0	0
Gentamicin	MIC_50_	32	0.38	0.75	0.38	0.25
	MIC_90_	>64	0.5	8	4	1
	Range	0.25–>256	0.25–0.75	0.38–12	0.19–6	0.094–3
	R(%)	68.8	0	25	5	0
Imipenem	MIC_50_	16	0.25	0.25	0.38	0.25
	MIC_90_	64	0.38	0.25	>32	0.25
	Range	0.38–>64	0.094–>32	0.19–0.38	0.25–>32	0.125–1.5
	R(%)	62.5	8.3	0	15	0
Meropenem	MIC_50_	8	0.25	0.25	0.75	0.5
	MIC_90_	>64	0.38	0.5	>32	0.75
	Range	0.25–>64	0.125–>32	0.19–0.5	0.25–>32	0.25–1.5
	R(%)	43.8	8.3	0	15	0
Tigecycline	MIC_50_	1	0.19	0.19	0.19	0.19
	MIC_90_	2	0.25	1.5	0.75	0.25
	Range	0.19–2	0.125–0.75	0.125–2	0.094–2	0.094–1
	R(%)^b^	ND	ND	ND	ND	ND
Tobramycin	MIC_50_	8	0.38	0.38	0.38	0.25
	MIC_90_	>64	0.38	2	1.5	0.38
	Range	0.38–96	0.19–0.38	0.25–3	0.19–2	0.094–1.5
	R(%)	56.3	0	0	0	0

*R(%), percentage of resistant isolates. MICs are expressed in μg/mL. ND, not determined; ^*a*^Clinical breakpoint according to CLSI guidelines; ^*b*^No clinical breakpoints available.*

Carbapenem-resistance was identified in three *A. pittii* isolates and one *A. lactucae* isolate, and was associated with the presence of genes encoding New Delhi metallo-β-lactamase (NDM) or OXA-type enzymes (Supplementary Data Sheet S1). *A. pittii* and *A. lactucae* isolates, however, tested susceptible to almost all other antimicrobial agents but ceftazidime, with rates of resistance of 5 and 25%, respectively. One *A. pittii* isolate was resistant to ciprofloxacin (MIC > 32 μg/mL) and another isolate of the same species was also resistant to gentamicin (MIC of 6 μg/mL). In contrast, *A. baumannii* isolates presented higher rates of resistance (75% for ceftazidime, 68.8% for ciprofloxacin and gentamicin, 62.5% for imipenem, 56.3% for tobramycin, 43.8% for meropenem, and 31.5% for amikacin), and resistance to carbapenems was usually associated with the production of OXA-type carbapenemases (Supplementary Data Sheet S1). Non-susceptibility to at least one agent in all but two or fewer antimicrobial categories, i.e., XDR (Magiorakos et al., 2012), was determined in 68.75% of *A. baumannii* isolates, the NDM-1 producing *A. pittii* was resistant to three or more antimicrobial agents from different categories, i.e., MDR and all other isolates were overall considered as highly susceptible. Finally, all the species of the Ab group showed low MIC values of tigecycline (Table 1) and, among all the isolates of the Ab group, only one *A. baumannii* isolate was resistant to colistin with an MIC of 8 μg/mL.

### Distribution of RND Efflux Pumps and Presence of Intrinsic *bla*_OXA_ Genes

**FIGURE 1:**
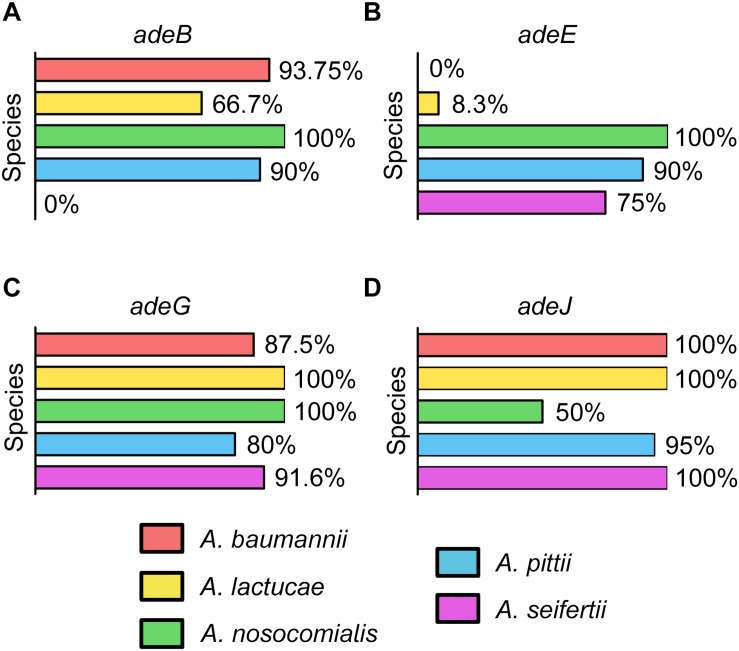
Occurrence of the genes encoding efflux pumps in the Ab group **(A)** *adeABC*, **(B)** *adeDE*, **(C)** *adeFGH*, and **(D)** *adeIJK*.

The occurrence of the different RND efflux pumps in the species of the Ab group is shown in Figures 1A–D. All isolates encoded at least one of the RND efflux systems known to be associated with antimicrobial resistance, and it was common to detect three or even four different efflux pumps in isolates from all *Acinetobacter* specie*s* (Supplementary Data Sheet S1). *adeG* and *adeJ* genes were identified in all species and were commonly present in most isolates (80 to 100 and 50 to 100%, respectively). *adeE* was highly disseminated within *A. nosocomialis* (100%) and *A. pittii* (90%), and was also present in 75% of *A. seifertii* isolates. However, *adeE* was found in only one isolate of *A. lactucae* and it was missing in *A. baumannii.* The *adeB* gene, on the other hand, was absent in *A. seifertii* but was ubiquitous in *A. baumannii* (93.75%) and *A. nosocomialis* (100%). Interestingly, isolates from *A. pittii* and *A. lactucae* carried a distinct *adeB*-like gene with 75 and 86% similarity to the *adeB* gene from *A. baumannii*, respectively. This gene was present in 90 and 66.7% of *A. pittii* and *A. lactucae* isolates, respectively.

In addition to the presence of different RND efflux pumps, we also evaluated the carriage of genes encoding intrinsic OXA enzymes. Intrinsic *bla*_OXA_ genes were reported in all *A. baumannii, A. lactucae* and 16 out of 20 *A. pittii* isolates but we were not able to detect them in any of the *A. nosocomialis* and *A. seifertii* isolates.

**FIGURE 2:**
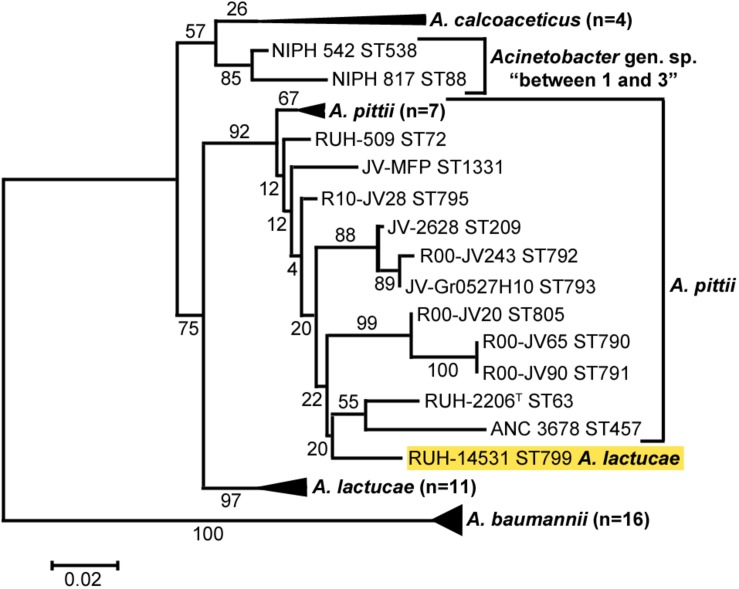
Cluster analysis of *A. baumannii*, *A. lactucae*, and *A. pittii* based on the partial amino acid sequence of their intrinsic OXA β-lactamases. Bootstrap values (%) are indicated in the branches. The scale bar indicates sequence divergence. The allele of *A. lactucae* clustering with those from *A. pittii* is highlighted in yellow. ST, sequence type; ND, not determined.

Cluster analysis of all sequences revealed that the OXA variants harbored by members of each of the three *Acinetobacter* species clustered together with high bootstrap values (Figure 2), the only exception being the intrinsic OXA β-lactamase of the *A. lactucae* RUH-14531 isolate that clustered together with OXA sequences from *A. pittii*.

### Biofilm Formation at 28 and 37°C

**FIGURE 3:**
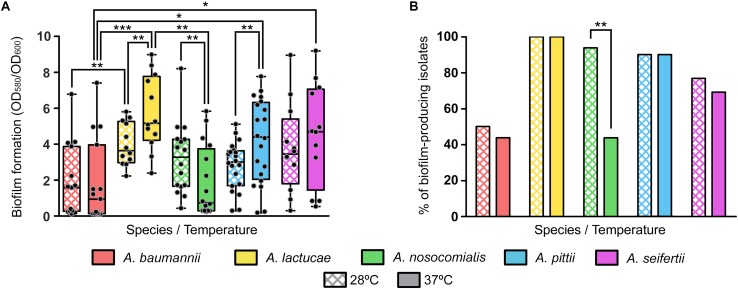
Biofilm formation at 28 and 37°C of the Ab group species. **(A)** Overlapping dot plot and box plot of the biofilm formation values of each species at 28 and 37°C. The boxes span from the first to the third quartile. The median is indicated as a segment inside the box. Whiskers indicate the minimum and maximum biofilm formation values. Each dot corresponds to the average biofilm formation value of an isolate after at least three biological replicates. Statistically significant differences upon temperature shift (Wilcoxon signed rank test) and between species (Kruskal–Wallis and *post hoc* tests) are highlighted with asterisks: (^∗^) if the *P*-value < 0.05, (^∗∗^) if the *P*-value ≤ 0.01, and (^∗∗∗^) if the *P*-value ≤ 0.001. **(B)** Percentage of biofilm-producing isolates of each species at 28 and 37°C. Isolates were considered as biofilm producers when the biofilm formation value was greater than 1. Statistically significant differences upon temperature shift (McNemar test) are highlighted with asterisks: (^∗^) if the *P*-value < 0.05 and (^∗∗^) if the *P*-value ≤ 0.01.

Biofilm formation values at both 28 and 37°C showed a high degree of variability among isolates of each of the five different *Acinetobacter* species included in the study (Figure 3A). No relevant differences in growth yields were observed among the different species or among members of the same species.

Nevertheless, statistically significant differences were observed between some species at each temperature; *A. lactucae* formed significantly more biofilm than *A. baumannii* at both temperatures and than *A. nosocomialis* at 37°C (Kruskal–Wallis test, *post hoc P*-value of 0.009, 0.001, and 0.004, respectively); and *A. pittii* and *A. seifertii* produced significantly more biofilm than *A. baumannii* at 37°C (Kruskal–Wallis test, *post hoc P*-values of 0.024 and 0.035, respectively). Despite the high dispersion of the data, and thus the lack of statistical significant differences for most pairwise comparisons, there were a few observations worth mentioning. *A. baumannii* isolates tended to form less biofilm than those of the other species, and biofilm formation was similar between *A. lactucae*, *A. pittii* and *A. seifertii*. Likewise, biofilm formation in *A. nosocomialis* resembled that of Ab group species other than *A. baumannii* at 28°C, but at 37°C it was comparable to that of *A. baumannii*.

Similar trends were observed when classifying isolates as biofilm producers and non-producers (Figure 3B). Our results showed that all *A. lactucae* isolates produced biofilm at both temperatures, and that the genetically related *A. pittii* species also presented a high proportion of biofilm-producing isolates (90%). In contrast, *A. baumannii* was the species with the least amount of biofilm-producing isolates, 50% or less, depending on the temperature. Also, upon shifting the temperature from 28 to 37°C, a reduction in the number of biofilm-producing isolates was observed in *A. baumannii*, *A. nosocomialis* and *A. seifertii*, while the number of biofilm-producing isolates remained unchanged in *A. lactucae* and *A. pittii*. Although this reduction was not statistically significant for *A. baumannii* and *A. seifertii* (from 50 to 43.8%, and from 83.3 to 75%, respectively), the effect of the temperature was greater in *A. nosocomialis*, in which shifting the temperature from 28 to 37°C decreased the percentage of biofilm-producing isolates from 93.8 to 43.8% (Figure 3B, McNemar test, *P*-value of 0.008). Thus, and under our experimental conditions, half of the isolates of this species were able to develop biofilm at 28°C but not at 37°C, and even in those isolates that did not lose the ability to produce biofilm at 37°C, a net reduction in the biofilm formation values was observed (Wilcoxon rank sum test, *P*-value of 0.01). On the other hand, the biofilm formation values of *A. lactucae* and *A. pittii* isolates were higher at 37°C than at 28°C (Wilcoxon rank sum test, *P*-value of 0.002 and 0.005, respectively; up to 2.3 fold changes, data not shown), although we did not detect biofilm non-producing isolates at 28°C turning into biofilm-producers at 37°C.

### Surface-Associated Motility Across Species of the Ab Group

**FIGURE 4:**
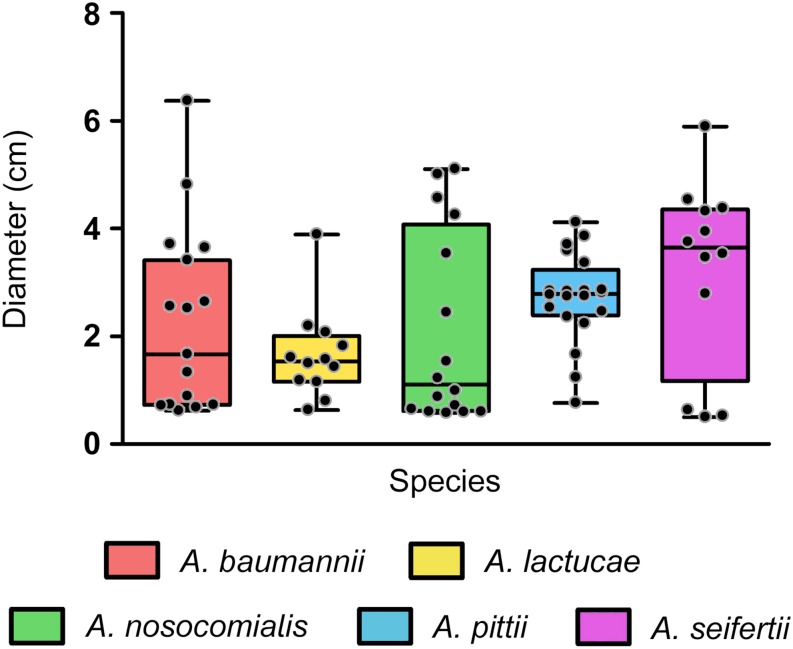
Surface associated motility in the Ab group species. Overlapping dot plot and box plot of the surface-associated motility of each species. Surface-associated motility was recorded as the diameter of growth after 18 h of incubation at 37°C using tryptone (5 g/L), NaCl (2.5 g/L), and agarose (0.3% w/v) motility media. The boxes span from the first to the third quartile. The median is indicated as a segment inside the box. Whiskers indicate the minimum and maximum diameters measured. Each dot corresponds to the average diameter measured for an isolate after at least four biological replicates. Statistically significant differences were not found (Kruskal–Wallis test, *P*-value > 0.05).

In our collection, surface-associated motility was observed in isolates of all the species studied, with greater intra-species variability for *A. baumannii*, *A. nosocomialis* and *A. seifertii* isolates (mean values ± SD (cm) of 2.315 ± 1.722, 2.078 ± 1.775, and 3.188 ± 1.755, respectively), than for *A. pittii* and *A. lactucae* (2.717 ± 0.829 and 1.653 ± 0.844, respectively) (Figure 4). Overall, differences in the distribution of surface-associated motility between species were not detected when taking into account the extent of motility. However, classification of the isolates into motile vs. non-motile according to the individual degree of motility showed higher rates of motility in *A. pittii* (95%) and *A. lactucae* (83.3%), followed by *A. seifertii* and *A. baumannii* (75 and 62.5%, respectively). *A. nosocomialis* was the least motile species of the group under the conditions tested with only half of the isolates being motile. Interestingly, rates of motility resembled those described above regarding biofilm formation at 37°C for *A. nosocomialis*. There was a positive correlation between motility and biofilm formation at 37°C in all species except for *A. pittii* (Spearman’s correlation coefficients between 0.622 and 0.7128, and *P*-values of 0.006 to 0.033), meaning that, in general, the more motile an isolate was, the more biofilm it tended to produce.

Interestingly, we also observed that all isolates displayed different surface-associated motility morphotypes, as previously described for *A. baumannii* (Skiebe et al., 2012), in a rather strain-specific than species-specific manner. A part from non-motile isolates (morphotype A), four additional different morphotypes (B, C, D and E, in order of abundance) were defined: morphotype B presented striated radiations from the inoculation point with well-defined edges; morphotype C radiated uniformly from the inoculation point and presented soft edges; morphotype D displayed densely-grown stria with thick edges; and morphotype E included the less abundant or unique morphotypes that could not be included in the any of the other groups (Supplementary Figure S1).

### Killing Assays Using the *C. elegans* Infection Model

**FIGURE 5:**
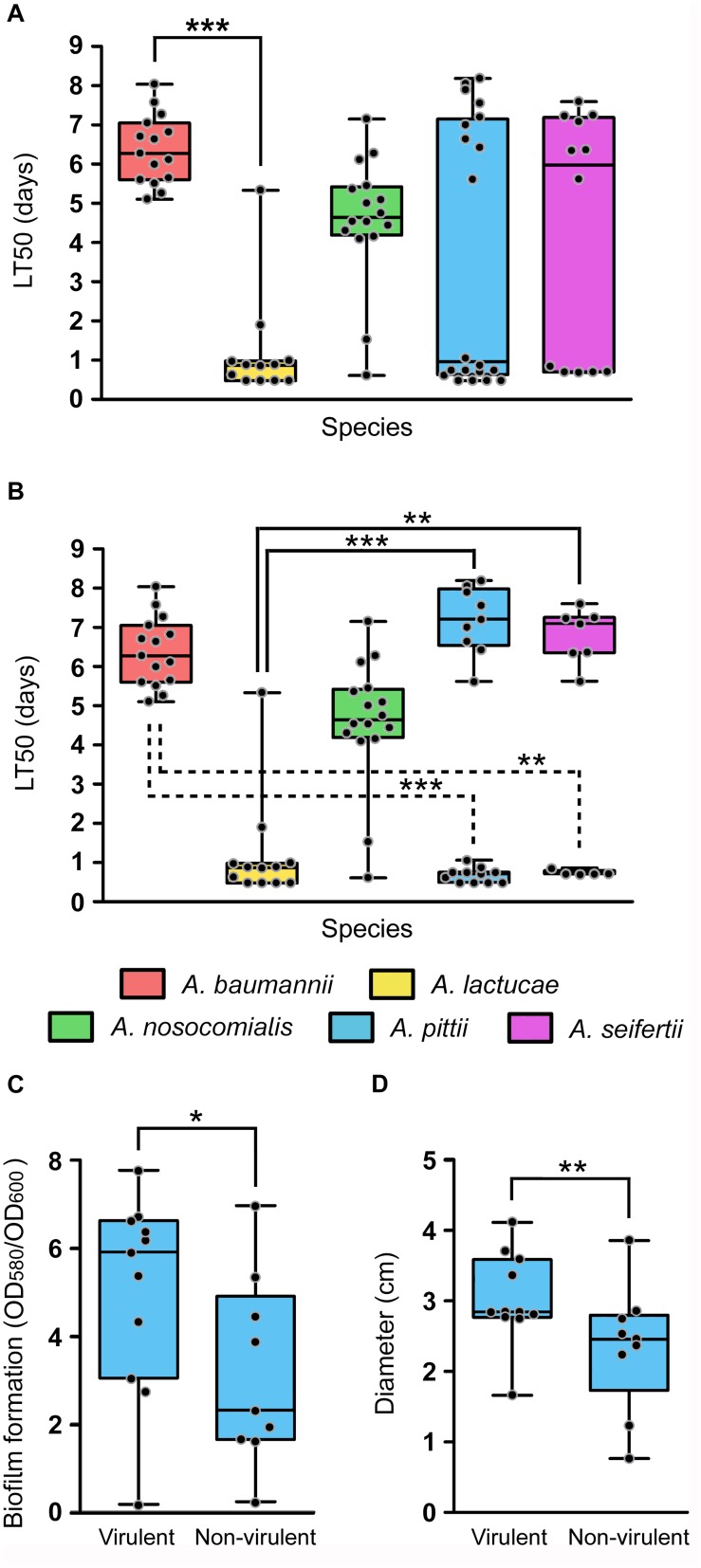
*In vivo* virulence of the Ab group species using the *C. elegans* infection model. Overlapping dot plot and box plot of **(A)** the overall LT50 values of each species; **(B)** the LT50 values of each species splitting *A. pittii* and *A. seifertii* in virulent (LT50 < 3 days) and non-virulent (LT50 > 3 days) subgroups; **(C)** the biofilm formation values at 28°C; and **(D)** the surface-associated motility values of *A. pittii* isolates divided into virulent and non-virulent subgroups. The boxes span from the first to the third quartile. The median is indicated as a segment inside the box. Whiskers indicate the minimum and maximum LT50 **(A,B)**, biofilm formation values **(C)** and diameter **(D)**. Each dot corresponds to the average phenotype value for an isolate after at least three biological replicates. Statistically significant differences between species (Kruskal–Wallis test) and subgroup species (Mann–Whitney *U*-test) are highlighted with asterisks: (^∗^) if the *P*-value < 0.05, (^∗∗^) if the *P*-value < 0.01, and (^∗∗∗^) if the *P*-value < 0.001.

The daily survival of the nematodes was scored during 15 consecutive days in order to obtain LT50 values of each isolate. Determination of the LT50 values presented, once more, ample intra-species variability (Figure 5A). Nevertheless, all isolates could be easily grouped into two distinct clusters according to their LT50 values. Those considered as virulent presented LT50 values ranging from 0.48 to 1.90 days and those included in the non-virulent group showed LT50 values ranging from 4.10 to 8.19 days. In view of these results, an LT50 cut-off value of 3 days was selected to differentiate virulent (LT50 < 3 days) from non-virulent isolates (LT50 > 3 days). All *A. baumannii* isolates were included within the non-virulent cluster, presenting LT50 values ranging from 4.85 to 8.04 days. *A. nosocomialis* was also shown to be an overall non-virulent species under this model, with 87.5% of isolates included in the non-virulent cluster and a median LT50 value of 4.65 days. On the other hand, according to this model, *A. lactucae* turned out to be the most virulent species (median LT50 of 0.871 days), with only one isolate included in the non-virulent group (Figure 5A). Of note, this was the only isolate of *A. lactucae* that carried a carbapenemase gene. In terms of virulence, *A. lactucae* presented significant differences in virulence only with *A. baumannii* (Kruskal–Wallis test, *post hoc P*-value < 0.001).

Interestingly, *A. pittii* and *A. seifertii*, when considered as a whole, showed very large dispersion of the data and presented no significant differences compared to either *A. baumannii* or *A. lactucae* (Figure 5A). However, *A. pittii* and *A. seifertii* isolates could be clearly divided into virulent (with LT50 values comparable to those of *A. lactucae*) and non-virulent subgroups (with LT50 values similar to those of *A. baumannii* and *A. nosocomialis*). When considered as independent groups, the virulent isolates of both *A. pittii* and *A. seifertii* presented significant differences in terms of virulence when compared to *A. baumannii* (Kruskal–Wallis test, *post hoc P*-value < 0.001 and 0.011, respectively), and the non-virulent isolates also presented significant differences when compared to *A. lactucae* (Kruskal–Wallis test, *post hoc P*-value < 0.001 and 0.001, respectively) (Figure 5B). Pairwise comparisons of the growth rates at 25°C between the different species showed that there were no differences in the growth rates between *A. baumannii* and *A. pittii/A. seifertii* (Student’s *t*-test, *P-*value 0.521 and 0.122, respectively). In addition, evaluation of the fitness rates at 25°C in all the species showed that there was no significant correlation between the LT50 values and the growth rates at 25°C of the isolates (Spearman’s correlation coefficient −0.349, *P*-value 0.12), and no differences in the growth rates at 25°C were found when the non-virulent and virulent isolates were compared as two independent groups (Student’s *t-*test, *P*-value 0.217), thus suggesting that the outcome of the killing assays was not affected by the growth rate of the isolates at 25°C.

In view of these results, we wondered if there were intra-species differences between the virulent and non-virulent subgroups of *A. pittii* and *A. seifertii* in regard to the other virulence-related phenotypes studied above, i.e., biofilm formation and motility, instead of considering these species as a whole. In *A. pittii*, isolates included within the virulent group were also shown to produce more biofilm at 28°C as well as to be more motile than those isolates included in the non-virulent group (Figures 5C,D, Mann–Whitney *U*-test, *P*-value of 0.038 and 0.002, respectively). In *A. seifertii*, however, it was not possible to associate virulent and non-virulent isolates with any other phenotype as there were no significant differences between both groups.

## Discussion

Despite the phenotypic similarities that hinder an accurate identification at the species level of the members of the Ab group using conventional identification methods, here we have sought any relevant differences among them in terms of antibiotic susceptibility patterns and virulence potential. Our efforts are aimed at reinforcing the idea that reporting and studying them collectively may be misleading in the clinical setting, as infections caused by different members of the Ab group might have substantially different clinical implications, as it has been noted for *A. pittii* and *A. nosocomialis* but has not yet been thoroughly examined for *A. lactucae* and *A. seifertii* (Chuang et al., 2011; Chusri et al., 2014).

In terms of antimicrobial resistance, results from our study are in good agreement with those of previous investigations reporting overall less susceptibility to most antimicrobial agents in *A. baumannii*, while *A. pittii* and *A. nosocomialis* tended to be more susceptible (Chuang et al., 2011; Lai et al., 2012; Chen et al., 2018; Pailhories et al., 2018). Studies that report on the antimicrobial susceptibility of *A. seifertii* or *A. lactucae*, on the other hand, are almost non-existent, since both species have only recently been described. Nevertheless, a study by Karah et al. (2011) included 3 *A. seifertii* isolates that were susceptible to all antibiotics tested. Another report that identified 28/287 bloodstream *A. seifertii* isolates detected two carbapenem-resistant isolates with a MDR phenotype, including resistance to colistin (Park et al., 2012). Although some authors have claimed that *A. seifertii* might be intrinsically resistant to polymyxins (Cayo et al., 2016; Narciso et al., 2017), we did not find colistin-resistant isolates of this species among our collection. Indeed, our results suggest that the novel members of the Ab group might be susceptible to most antibiotics, resembling other non-*baumannii Acinetobacter* species.

Another characteristic of many *Acinetobacter* species is the carriage of naturally occurring oxacillinases, such as OXA-51 in *A. baumannii*, and it is widely accepted that members of the same *Acinetobacter* species seem to harbor OXA allelic variants that belong to the same OXA group (Perichon et al., 2014). Such intrinsic OXA groups may account for β-lactam resistance, especially when insertion sequences are found upstream from the *bla*_OXA_ gene (Higgins et al., 2010a). Nevertheless, bacterial isolates belonging to *A. nosocomialis* and *A. seifertii* might be an exception since no intrinsic OXAs have yet been found in the genome sequences of *A. nosocomialis* and *A. seifertii* (Perichon et al., 2014), except for the presence of plasmid-borne intrinsic OXA from *A. baumannii* (Lee et al., 2012). In our study, also in good agreement with data from Perichon et al. (2014), we confirmed the presence of species-specific intrinsic OXA genes in *A. baumannii*, *A. lactucae* and *A. pittii*, and the lack of them in *A. nosocomialis* and *A. seifertii* (Figure 2).

The prevalence of *adeB* in *A. baumannii* from our collection was high (93.75%) albeit within the range of previous reports (53–97%) (Chu et al., 2006; Nemec et al., 2007; Lin et al., 2009; Rajamohan et al., 2010; Modarresi et al., 2015; Nowak et al., 2015), but we acknowledge that isolates of *A. baumannii* in our study are the least polyclonal of all five species (Supplementary Data Sheet S1), although it is not clear what was the degree of clonality in previous studies. Interestingly, the *adeB* gene was present in all the *A. nosocomialis* isolates as well as in many *A. lactucae* and *A. pittii* isolates, confirming that this efflux system is not restricted to *A. baumannii* (Espinal et al., 2011). Likewise, the *adeIJK* and *adeFGH* operons were previously considered as being highly specific of *A. baumannii*, and *adeDE* as being specific of *A. pittii*. Here we have shown that *adeJ* and *adeG* genes are present in all species, and that *adeE* was present in all *A. nosocomialis* isolates and was detected among *A. seifertii* and *A. lactucae*, although it was missing in *A. baumannii*. Hou et al. (2012) reported a small number of *A. baumannii* isolates harboring *adeE* together with *adeB*, albeit species identification was performed with phenotypic methods which we now know may lead to misidentification.

The study of virulence-related phenotypes revealed that biofilm formation and surface-associated motility remained highly variable among isolates from the same species. Indeed, several authors have pointed out that the variability observed in biofilm formation might be due to this phenotype being clone-specific (Rodriguez-Baño et al., 2008; Wroblewska et al., 2008; de Breij et al., 2010; Giannouli et al., 2013; Kaliterna and Goic-Barisic, 2013; Gentile et al., 2014), unfortunately, there is still little knowledge regarding the population structure of *Acinetobacter* species other than *A. baumannii* and thus, it is not possible to compare major clonal groups for each of these species. In addition, and as previously described for *A. baumannii* (McQueary et al., 2012), we observed that motile isolates displayed different surface-associated morphotypes, which were not species-dependent but, again, strain-specific. Nonetheless, despite the intra-species variability observed, we were still able to find overall inter-species differences. In fact, the ability to produce biofilm not only differed between species but it was also temperature-dependent. Temperature modulation of biofilm formation, as well as motility and antibiotic resistance, have been previously observed in *A. baumannii* ATCC 17978, although the underlying regulatory mechanisms remain unclear (De Silva et al., 2018). Our results suggest different thermoregulation strategies of biofilm formation between isolates of *A. baumannii*, *A. nosocomialis*, and *A. seifertii* and those of *A. lactucae* and *A. pittii*.

Infection assays using the *C. elegans* animal model also revealed overall differences between isolates of *A. baumannii* and *A. nosocomialis*, being mostly non-virulent, and those of *A. lactucae*, being highly virulent, although statistical significance was only achieved between *A. baumannii* and *A. lactucae*. The infection assays highlight the existence of two subpopulations within our collection of *A. pittii* and *A. seifertii* isolates. It remains unclear whether this observation represents the intrinsic heterogeneity within each species, or it is an indication that the taxonomic delineation for these two species needs to be further investigated. It is worth mentioning that *A. lactucae* isolates were previously classified as “*A. pittii*-like” (Espinal et al., 2015), since both species are closely related, and the *A. pittii* taxon seems to be taxonomically difficult as it contains several related (“-like”) strains with no clear phenotypic/genotypic discontinuities (Perichon et al., 2014; Mari-Almirall et al., 2017). It is also plausible that our results reflect a process of adaptation to a particular ecological niche, such as the human host.

To summarize, we observed that species which are more related to the nosocomial environment, i.e., *A. baumannii* and *A. nosocomialis*, had non-virulent phenotypes assessed in the *C. elegans* infection model and tended to form less biofilm than the other species. In contrast, *A. pittii*, which is more ecologically diverse (Al Atrouni et al., 2016), showed greater virulence, together with the two recently described species *A. lactucae* and *A. seifertii* (Nemec et al., 2015; Mari-Almirall et al., 2017). Interestingly, the only study that so far attempted to compare the virulence traits of the different species of the Ab group (*A. lactucae* was not included) also reported that *A. seifertii* isolated from bloodstream infections showed enhanced *in vitro* virulence properties (Na et al., 2016).

These findings however, do not seem to correlate with clinical studies, where infections caused by *A. baumannii* in particular but also by *A. nosocomialis* seem to be associated with a less favorable outcome than that of patients with infections caused by other species (Lee et al., 2011; Wisplinghoff et al., 2012; Chusri et al., 2014; Lee et al., 2014; Fitzpatrick et al., 2015). There are a few studies that suggest that such observation might not be due to a higher pathogenicity of the former species and rather be the consequence of inappropriate early empirical treatment, since a higher proportion of *A. baumannii* infections are caused by MDR strains.

The underlying question we intended to address here is whether the novel *Acinetobacter* species present a higher pathogenicity potential than *A. baumannii* that may be currently masked by their overall antimicrobial susceptibility. If so, should we expect worse clinical outcomes upon the acquisition of resistance by these *Acinetobacter* species? Under this assumption, *A. nosocomialis* and *A. baumannii* might have suffered a reduction of intra-species diversity (population bottleneck) resulting from adaptation to the human host and/or antibiotic selection, while the variability observed among isolates of *A. pittii*, *A. seifertii* and *A. lactucae* might reflect their emergence and distinct degrees of adaptation as human nosocomial pathogens (Schleicher et al., 2013; Jain et al., 2016; Chen et al., 2018; Pailhories et al., 2018; Rocha et al., 2018). Nevertheless, it is important to acknowledge that the results provided in our study arise from the usage of *in vitro* and nematode infection models that do not entirely reflect more complex clinical scenarios and, thus, the extrapolation of these results to infer virulence in the human host must be taken with extreme caution. For instance, respiratory tract infections and in particular both community-acquired as well as nosocomial pneumonia are mainly attributed to *A. baumannii* and *A. nosocomialis* while there is little evidence that the other species are capable of causing such infections in humans (Wang et al., 2013; Larcher et al., 2017).

With the recent advances in bacterial identification methods it is likely that the number of reported infections caused by the novel species of *Acinetobacter* increases over time. This increase should provide more robust clinical data to evaluate the characteristics of infections caused by the different members of the Ab group and reveal their pathogenic potential in the human host.

## Data Availability Statement

The authors state that the dataset and partial protein sequences supporting the findings of this study are available within the article and its Supplementary Material.

## Author Contributions

CC, LR, PH, HS, IR, and JV collected the strains. CC, HS, IR, and JV conceived and designed the experiments. CC, CR, MM-A, LR, and PH performed the experiments. CC, CR, MM-A, PH, HS, IR, and JV analyzed and interpreted the data. CC, PH, HS, IR, and JV wrote the manuscript. All authors critically revised the manuscript for intellectual content and read and approved the final manuscript.

## Conflict of Interest

The authors declare that the research was conducted in the absence of any commercial or financial relationships that could be construed as a potential conflict of interest.

## Footnotes

1 http://www.bacterio.net/acinetobacter.html
