# Supplementary figures and images for: In vitro and in vivo Virulence Potential of the Emergent Species of the Acinetobacter baumannii (Ab) Group
Source: Front Microbiol. 2019 Oct 24;10:2429. doi: 10.3389/fmicb.2019.02429 (PMC6821683; doi:10.3389/fmicb.2019.02429)

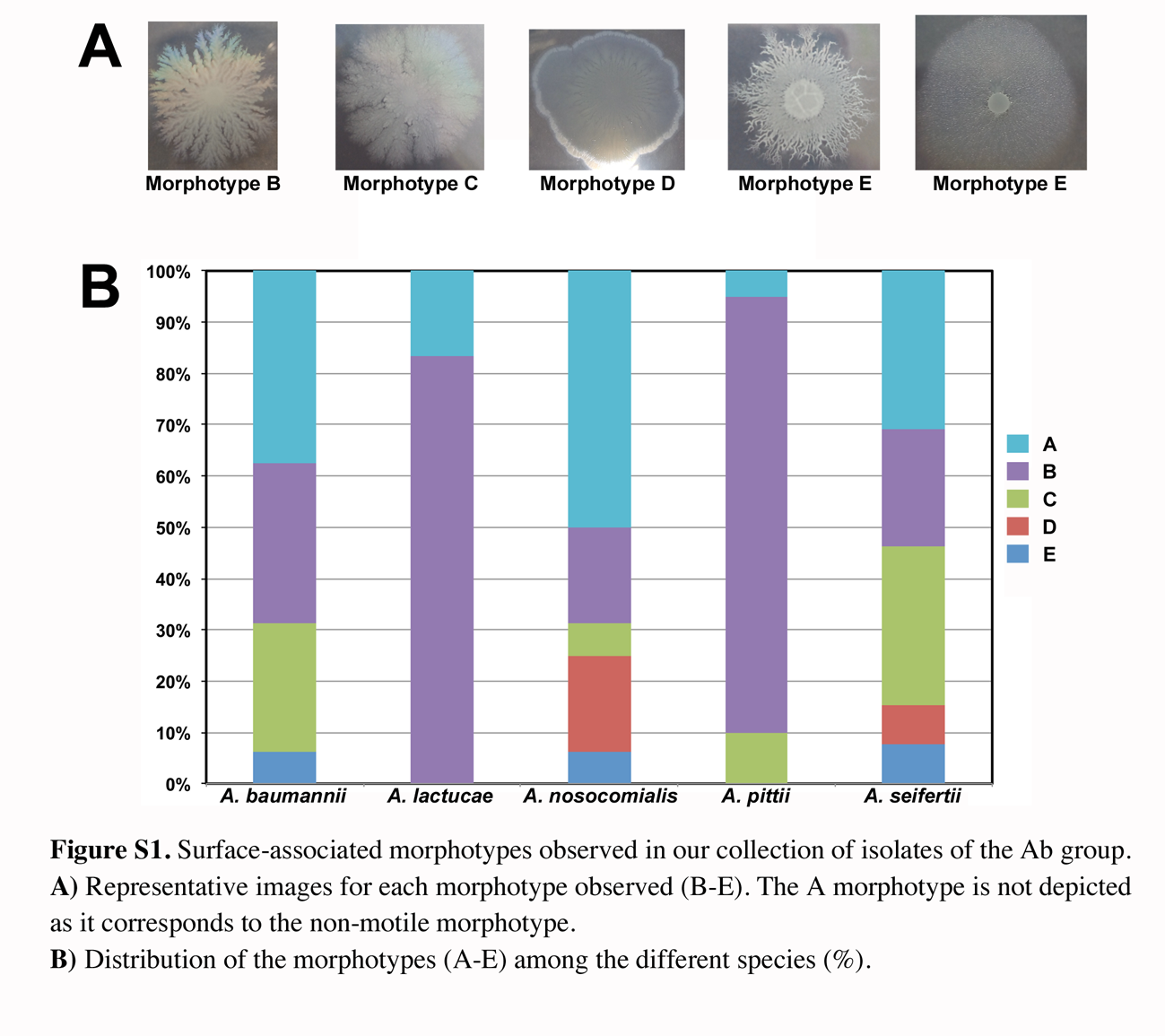

Supplement: Supplementary file 3 [file Image_1.tif]
