# Supplementary material for: In vitro and in vivo Virulence Potential of the Emergent Species of the Acinetobacter baumannii (Ab) Group
Source: Front Microbiol. 2019 Oct 24;10:2429. doi: 10.3389/fmicb.2019.02429 (PMC6821683; doi:10.3389/fmicb.2019.02429)
Supplement: Supplementary file 4 [file Table_1.pdf]

Table S1. Primers used in this study. Boxes in dark grey indicate the primer combination used to obtain the results shown in this report for each species; boxes in light gray indicate that the primers were employed, but that amplification was inefficient or absent. Abau, *A. baumannii*; Alac, *A. lactucae*; Ansc, *A. nosocomialis*; Apit, *A. pittii*; Asei, *A. seifertii*.

Gene, Name	Sequence (5' - 3')	Species				
adeB		Abau	Alac	Ansc	Apit	Asei
adeBUp	ATGTCACAATTTTTATTCGTCGTC	dark grey	light grey	dark grey	dark grey	light grey
adeBLw	TTAGGATGAGATTTTTTTCTTAGAGG	dark grey	light grey	dark grey	light grey	light grey
O3[a]	GTATGAATTGATGCTGC	dark grey	light grey	dark grey	light grey	light grey
O4[a]	CACTCGTAGCCAATACC	dark grey	light grey	dark grey	light grey	light grey
adeB 2_F[b]	GAATAAGGCACCGCAACAAT	dark grey	dark grey	dark grey	dark grey	light grey
adeB 2_R[b]	TTTCGCAATCAGTTGTTCCA	dark grey	dark grey	dark grey	dark grey	light grey
adeB-pitLw	GTGCAGTATCGTAAGGAACGC				dark grey	
adeB_dijUp	CGTGTTGAAGGTGTTGGTAAAGT		dark grey			
adeB_dijLw	ATGCAGGCATACCTAAGTTCGC		dark grey			
adeE						
adeEUp	GATGACGCGATTGTCGTTG	dark grey	dark grey	dark grey	dark grey	dark grey
adeELw	GGATCCGCACGAGCAATCACAGCTTC	dark grey	dark grey	dark grey	dark grey	light grey
adeELw2	CCTGCTCCTGTAGATACAGC					dark grey
adeG						
adeGUp	GTTGCTCGTGTCGAACTTGC	dark grey	dark grey	dark grey	dark grey	dark grey
adeGLw	AGGAACGAAACCACCTGGAAC	dark grey	dark grey	dark grey	dark grey	dark grey
adeJ						
adeJUp	CTGGCTTATGACACGACTC	dark grey	dark grey	dark grey	dark grey	dark grey
adeJLw	GGATCCCCATACCACGCTGAATCAATG	dark grey	dark grey	dark grey	dark grey	dark grey
Intrinsic OXA						
OXA69-F	GATCTACTCAAGTTACATTAATTAGC	dark grey				
OXA69-R	AGTGAACTGGGCTATAAAC	dark grey				
OXA51nF[c]	TAATGCTTTGATCGGCCTTG		light grey	light grey	light grey	light grey
OXA51nR[c]	TGGATTGCACTTCATCTTGG		light grey	light grey	light grey	light grey
OXA_G3_Up	GCTCTTTTCCTTGCTATTAGTAC		dark grey		dark grey	
OXA_G3_Lw	TACCTAGCTGTTCTAATCC		dark grey		dark grey	

[a] Designed by Magnet S., Courvalin P., Lambert T. (2001). Resistance-nodulation-cell division-type efflux pump involved in aminoglycoside resistance in *Acinetobacter baumannii* strain BM4454. *Antimicrob. Agents Chemother.* 45, 3375-3380. [b] Designed by Nowak J., Seifert H., Higgins P.G. (2015). Prevalence of eight resistance-nodulation-division efflux pump genes in epidemiologically characterized *Acinetobacter baumannii* of worldwide origin. *J. Med. Microbiol.* 64, 630-635. doi: 10.1099/jmm.0.000069. [c] Designed by Woodford *et al.* (2006). Multiplex PCR for genes encoding prevalent OXA carbapenemases in *Acinetobacter* spp. Int. J. Antimicrob. Agents 27, 351-353. doi: 10.1016/j.ijantimicag.2006.01.004.
